# Supplementary material for: Antibody signatures against viruses and microbiome reflect past and chronic exposures and associate with aging and inflammation
Source: iScience. 2024 May 16;27(6):109981. doi: 10.1016/j.isci.2024.109981 (PMC11167443; doi:10.1016/j.isci.2024.109981)
Supplement: Document S1. Results [file mmc1.pdf]

iScience, Volume *27*

Supplemental information

Antibody signatures against viruses and microbiome reflect past and chronic exposures and associate with aging and inflammation

Sergio Andreu-Sánchez, Aida Ripoll-Cladellas, Anna Culinscaia, Ozlem Bulut, Arno R. Bourgonje, Mihai G. Netea, Peter Lansdorp, Geraldine Aubert, Marc Jan Bonder, Lude Franke, Thomas Vogl, Monique G.P. van der Wijst, Marta Melé, Debbie Van Baarle, Jingyuan Fu, and Alexandra Zhernakova

Supplementary Material

Results

Antibody responses against rhinoviruses are correlated with longer telomeres and cellular composition changes

We explored the observed association of antibodies against rhinovirus and longer telomeres. Due to the lack of a clear selection criterion for a single representative rhinoviral peptide, we employed a regularized linear model (lasso penalty) regression on different bootstraps of the complete dataset to identify rhinoviral peptides that were consistently associated with different telomere lengths (TLs). Our analysis revealed that one individual peptide, *twist_35344*, targeting 'polyprotein & VP1 capsid protein' and a score representing the breadth of antibody-bound peptides of rhinoviral origin, total number of anti-rhinovirus antibodies, were frequently selected as features in the regularized model (median among cell types of 93% and 75.8%, respectively). We then compared TLs from all individuals with the breadth of antibody-bound rhinoviral proteins and *twist_35344* and found that TLs were more strongly associated with the breadth of antibody-bound rhinoviral proteins than with *twist_35344* (linear-mixed model of the effect on all TL, $P_{breadth\_antibody}=1.8x10^{-11}$, $P_{twist\_35344}=1.38x10^{-5}$). Consequently, we utilized the count score representing the breadth of rhinoviral antibodies to investigate the associations with TL.

It is worth noting that rhinoviral antibody-bound peptides are often observed in younger individuals (1,2), while cytomegalovirus (CMV) infections are more prevalent at older age. To address the potential confounding effect of age, we adjusted for CMV status and compared the strength of the association with and without adjustment for age (which was treated as a categorical variable to account for possible non-linear effects). Remarkably, the association between the breadth of rhinoviral antibodies and TL remained significant after adjusting for age (linear-mixed model of the effect on all TL, average effect breadth rhinovirus antibodies

in all TLs without accounting for age=2.688x10^{-2}, P=5.82x10^{-10}; average effect breadth rhinovirus antibodies in all TLs accounting for age effect=1.677x10^{-2}, P=2.70x10^{-5}), indicating that the association of the presence of rhinoviral antibodies to longer telomeres is partially independent of participant age. Similarly, after matching 393 participants with antibody responses against *twist_35344* (rhinoviral ‘polyprotein & VP1 capsid protein’) with those with no antibody response, based on the nearest age-sex match (see **Methods**), we still identified significant positive effects of rhinovirus on TLs (multivariable model with age, CMV and *twist_35344,* $effect_{matched}$=0.21, $P_{matched}$=2.5x10^{-4}, $effect_{notmatched}$=0.173, $P_{notmatched}$=4.27x10^{-4}). Furthermore, we investigated the independence of the rhinoviral association from smoking, as smoking is often associated with increased rhinoviral infections and typically considered a factor negatively associated with TL. Our analysis revealed that the association between the breadth of rhinoviral antibodies and TL remained significant after adjusting for smoking (effect on all TL, effect=1.841x10^{-2}, P=4.61x10^{-6}).

Overall, after accounting for CMV, smoking habits, age and sex, the breadth of rhinoviral antibodies was associated with all TLs (P<1.5x10^{-03}), but this effect was significantly different between cell types (likelihood ratio test (LRT) model with interaction term of cell type and rhinovirus vs model without, P=8.576x10^{-7}). With respect to TLs, rhinoviruses were more strongly associated with the TLs of memory T-cells (estimate=0.02, P=4.05x10^{-6}), lymphocytes (estimate=0.019, P=3x10^{-5}) and naïve T-cells (estimate=0.019, P=4.61 x10^{-5}) [**Table S4**]. We did not find statistical significance supporting differences between age groups (LRT model with age group interaction with rhinovirus vs model without, P=0.46).

We also observed that the breadth of rhinoviral antibody responses was significantly associated with specific cell populations, mirroring those attributed to CMV, but in the opposite direction [**Table S4**]. For example, we found significant associations between rhinoviral antibody breadth and intermediate monocytes (CD14+CD16+) (effect=-1.6x10^{-3}, P=1.1x10^{-4}), CD8+ naïve cells (effect=1.29x10^{-3}, P=7.4x10^{-4}) and proliferative CD4+ Treg cells (effect=9.1x10^{-4}, P=1.3x10^{-3}), among others [**Fig S1A**]. To explore the mediating role of

cell composition and TL in these associations, we conducted a mediation analysis that included both CMV infection and the breadth of rhinovirus antibodies. The results suggest that CMV and rhinoviral effects are independent and that cell composition partially mediated the changes in TL [**Fig S1B**]. Specifically, the effect of rhinovirus on TL in naïve T-cells was found to be partially mediated by the predicted cell counts of CD8+ naïve cells, accounting for 17.1% (95% CI, 0.07–0.35) of its effect on TL.

Cell composition association to CMV serostatus in single-cell data

In our previous results using measured cell counts and cell counts predicted from bulk RNA-seq, we found a CMV-associated expansion of CD8+ T-cells, particularly CD8+ EM, and a decrease of proliferative and naïve CD4+ T-cells. Using scRNA-seq data, we used both the low (l1)- and high (l2)-resolution cell-type-annotations predicted by Azimuth (3) to classify cells in order to closely reflect the resolution of the measured and deconvoluted blood cell counts (see **Methods**). At l1 level, we replicated the previously observed association [**Fig 3B**] between CD8+ T-cells and CMV serostatus (effect=0.74, P=$4x10^{-7}$, FDR=$3.2x10^{-6}$) [**Fig S2A**]. At l2 level, we identified four significant associations (FDR<0.05) [**Fig 4A**] and replicated three previously observed cell proportion–CMV associations [**Fig 3B**]: the negative association of CMV with CD4+ naïve T-cells (effect=-0.56, P=$3.7x10^{-3}$, FDR=$2.2x10^{-2}$), which were previously reported to be reduced by CMV (4), a positive association between CMV and CD8+ effector memory T-cells (TEM) (effect=0.95, P=$32.3x10^{-6}$, FDR=$2.7x10^{-5}$), a subpopulation able to expand and generate TEFF cells upon rechallenge (5); and a significant decrease of regulatory T-cells (Treg) (effect=-0.52, P=$2.2x10^{-3}$, FDR=$1.8x10^{-2}$), with such a reduction previously observed in CMV-positive males (6). We found an additional association that was not seen in the deconvoluted data: an increase of CD4+ cytotoxic T lymphocytes (CTL) (effect=2.66, P=$8.5x10^{-12}$, FDR=$2.1x10^{-10}$) with CMV seropositivity, with CTL known to be mediators of antiviral defense (7) [**Table S5A**].

CMV infection linked with depletion and overexpression of transcriptional pathways in CD4+ CTL and CD8+ TEM cells

We assessed whether our reported DEGs belonged to similar functional pathways, thereby highlighting the biological interplay between CMV seropositivity and gene expression [**Fig 4E**]. To explore this, we performed a functional enrichment analysis separately for the up- and down-DEGs in each of the two cell subtypes [**Table S5D**]. Within the genes positively associated with CMV serostatus in the CD4+ CTL cells, we found an enrichment of the pathways *negative regulation of metabolic* (GO:0009892, enrichment ratio=1.55, $P=2.2x10^{-16}$, $FDR=2.2x10^{-16}$) and *negative regulation of gene expression* (GO:0010629, enrichment ratio=1.75, $P=2.2x10^{-16}$, $FDR=2.2x10^{-16}$). In addition, we found an enrichment for *translation* (GO:0006412, enrichment ratio=2.34, $P=2.2x10^{-16}$, $FDR=2.2x10^{-16}$) and *peptide biosynthetic process* (GO:0043043, enrichment ratio=2.3, $P=2.2x10^{-16}$, $FDR=2.2x10^{-16}$). Unlike the many viruses that suppress cellular protein synthesis, CMV stimulates host mRNA translation and polyribosome formation, even in uninfected cells (8). Conversely, we found a down-regulation of several lipid biosynthetic processes, including *lipid biosynthetic process* (GO:0008610, enrichment ratio=1.27, $P=1.2x10^{-6}$, $FDR=3.6x10^{-3}$), *phospholipid biosynthetic process* (GO:0008654, enrichment ratio=1.35, $P=1.4x10^{-5}$, $FDR=2x10^{-2}$) and *glycerolipid biosynthetic process* (GO:0045017, enrichment ratio=1.36, $P=2.7x10^{-5}$, $FDR=2x10^{-2}$). Systemic metabolic sequelae such as insulin resistance and dyslipidemia represent long-term health consequences of many infections (e.g., human immunodeficiency virus, hepatitis C virus and SARS-CoV-2) (9). In addition, the *anion transport* pathway (GO:0006820, enrichment ratio=1.3, $P=2.7x10^{-5}$, $FDR=2x10^{-2}$), which can be modulated by viral proteins (10), was negatively associated with CMV seropositivity. On the other hand, focusing on CD8+ TEM cells, many immune-related pathways, such as *regulation of leukocyte activation* (GO:0002694, enrichment ratio=3.21, $P=2.2x10^{-6}$, $FDR=1.9x10^{-3}$) and *immune effector process* (GO:0002252, enrichment ratio=2.57, $P=1.7x10^{-8}$, $FDR=1.3x10^{-4}$), were positively associated with CMV seropositivity, together with *exocytosis* (GO:0006887, enrichment

ratio=2.52, P=$3.3x10^{-6}$, FDR=$2.5x10^{-3}$), one of the major mechanisms of cytotoxicity involved in the clearance of virus-infected cells (11). In addition, our set of negatively enriched pathways revealed a regulation of signaling receptor activity, specifically the *G protein-coupled receptor signaling* pathway (GO:0007186, enrichment ratio=1.97, P=$2.9x10^{-8}$, FDR=$1.1x10^{-4}$). CMV, as a member of the *Herpesviridae* family, encodes G protein-coupled receptors (GPCRs) showing homology to human chemokine receptors, which might be used as decoy receptors to prevent cytokine action (12). By means of these constitutive GPCRs, herpesviruses have devised strategies to rewire host cell-signaling pathways, thereby promoting viral biology and subsequent pathogenic effects (13). Lastly, since most of the pathways were subpopulation-specific, we explored the enriched pathways among the DEGs shared between CD4+ CTL and CD8+ TEM cells. Besides the positive association of leukocyte activation and immune effector processes with CMV seropositivity, we found the *homotypic cell-cell adhesion* pathway (GO:0034109, enrichment ratio=14.63, P=$2.1x10^{-5}$, FDR=$2.1x10^{-2}$) to be enriched in our set of shared DEGs. Indeed, an up-regulation of adhesion molecules has been reported to occur on activated T-cells by culture with the CMV antigen, mainly on CD45RO+ T memory cells, which may have a role in immune reaction or inflammatory modulation (14).

References

1. Winther B, Gwaltney Jr JM, Mygind N, Hendley JO. Viral-induced rhinitis. Am J Rhinol. 1998;12(1):17–20.

2. Andreu-Sánchez S, Bourgonje AR, Vogl T, Kurilshikov A, Leviatan S, Ruiz-Moreno AJ, et al. Phage display sequencing reveals that genetic, environmental, and intrinsic factors influence variation of human antibody epitope repertoire. Immunity. 2023 May 9.

3. Hao Y, Hao S, Andersen-Nissen E, Mauck 3rd WM, Zheng S, Butler A, et al. Integrated analysis of multimodal single-cell data. Cell. 2021 Jun;184(13):3573–3587.e29.

4. Manusama O, Singh S, Brooimans RA, Wijkhuijs A, van der Ent M, Drexhage HA, et al. Reduced numbers of naïve CD4 + T cells and an altered CD4/CD8 balance in depressed common variable immune deficiency (CVID) patients. Is thymosin-α1 a possible treatment? Int Immunopharmacol. 2023 Jun 1;119:110168.

5. Surh CD, Sprent J. Homeostasis of naive and memory T cells. Immunity. 2008 Dec 19;29(6):848–62.

6. Van Der Heiden M, Van Zelm MC, Bartol SJW, De Rond LGH, Berbers GAM, Boots AMH, et al. Differential effects of Cytomegalovirus carriage on the immune phenotype of middle-aged males and females. Scientific Reports 2016 6:1. 2016 May 31;6(1):1–12.

7. Verma S, Weiskopf D, Gupta A, McDonald B, Peters B, Sette A, et al. Cytomegalovirus-Specific CD4 T Cells Are Cytolytic and Mediate Vaccine Protection. J Virol. 2016 Jan 1;90(2):650.

8. Mckinney C, Zavadil J, Bianco C, Shiflett L, Brown S, Mohr I. Global Reprogramming of the Cellular Translational Landscape Facilitates Cytomegalovirus Replication. 2014

9. Palmer CS. Innate metabolic responses against viral infections. Nature Metabolism 2022 4:10. 2022 Oct 20;4(10):1245–59.

10. Charlton FW, Pearson HM, Hover S, Lippiat JD, Fontana J, Barr JN, et al. Ion Channels as Therapeutic Targets for Viral Infections: Further Discoveries and Future Perspectives. Viruses. 2020 Aug 1;12(8).

11. Smyth MJ, Trapani JA. The Relative Role of Lymphocyte Granule Exocytosis versus Death Receptor-Mediated Cytotoxicity in Viral Pathophysiology. J Virol. 1998 Jan;72(1):1.

12. Hernaez B, Alcamí A. Virus-encoded cytokine and chemokine decoy receptors. Curr Opin Immunol. 2020 Oct 1;66:50–6.

13. Van Senten JR, Bebelman MP, Fan TS, Heukers R, Bergkamp ND, Van Gasselt P, et al. The human cytomegalovirus-encoded G protein–coupled receptor UL33 exhibits oncomodulatory properties. J Biol Chem. 2019 Nov 11;294(44):16297.

14. Ito M, Watanabe M, Sakurai M, lhara T, Kamiya H. Increased Expression of Adhesion Molecules (CD54, CD29 and CD44) on Fibroblasts Infected with Cytomegalovirus. Microbiol Immunol. 1995 Feb 1;39(2):129–33.

15. Seidler S, Zimmermann HW, Bartneck M, Trautwein C, Tacke F. Age-dependent alterations of monocyte subsets and monocyte-related chemokine pathways in healthy adults. BMC Immunol. 2010 Jun 21;11:30.

16. Li M, Yao D, Zeng X, Kasakovski D, Zhang Y, Chen S, et al. Age related human T cell subset evolution and senescence. Immunity and Ageing. 2019 Sep 11;16(1):1–7.

17. Shao C, Zhu C, Zhu Y, Hao J, Li Y, Hu H, et al. Decrease of peripheral blood mucosal-associated invariant T cells and impaired serum Granzyme-B production in patients with gastric cancer. Cell Biosci. 2021 Dec 1;11(1):1–9.

18. Seirafian S, Prod'Homme V, Sugrue D, Davies J, Fielding C, Tomasec P, et al. Human cytomegalovirus suppresses Fas expression and function. J Gen Virol. 2014;95(Pt 4):933.

19. Reitsma JM, Savaryn JP, Faust K, Sato H, Halligan BD, Terhune SS. Antiviral Inhibition Targeting the HCMV Kinase pUL97 Requires pUL27-Dependent Degradation of Tip60 Acetyltransferase and Cell-Cycle Arrest. Cell Host Microbe. 2011 Feb 17;9(2):103–14.

20. van der Wijst MGP, Brugge H, de Vries Dylan H and Deelen P, Swertz MA, LifeLines Cohort Study, BIOS Consortium, et al. Single-cell RNA sequencing identifies celltype-specific cis-eQTLs and co-expression QTLs. Nat Genet. 2018 Apr;50(4):493–7.
