# Supplementary material for: Antibody signatures against viruses and microbiome reflect past and chronic exposures and associate with aging and inflammation
Source: iScience. 2024 May 16;27(6):109981. doi: 10.1016/j.isci.2024.109981 (PMC11167443; doi:10.1016/j.isci.2024.109981)
Supplement: Document S2. Figures S1–S3 [file mmc2.pdf]

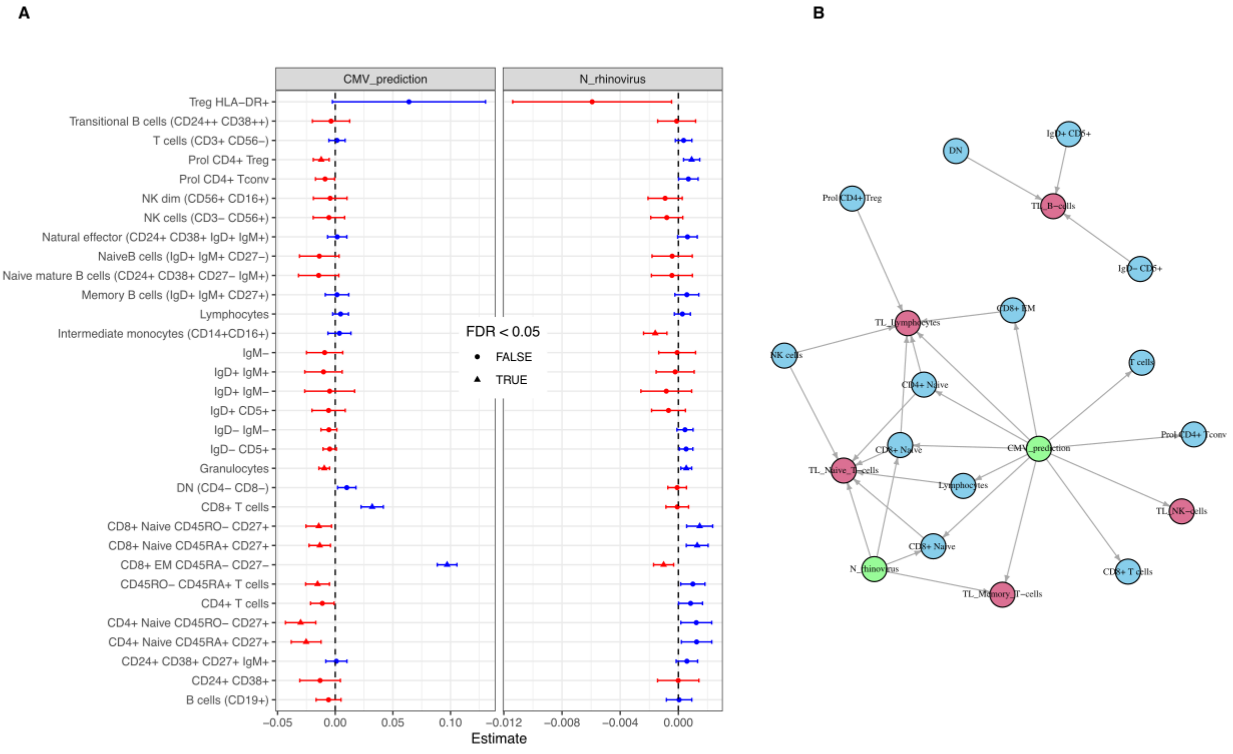

Figure S1. Rhinovirus and CMV signal on cell composition, related to Figure 3. A. Estimated effect size of CMV prediction and breadth of rhinovirus peptides in deconvoluted cell populations (ALR-normalized), controlled for age and sex. Color indicates the direction of the effect (red: negative, blue: positive). Triangles indicate $FDR_{BH} < 0.05$ associations. Circles indicate an $FDR_{100perm} \geq 0.05$. Error bars represent 95% confidence interval from the estimate. **B.** Mediation network built using Regmed. Green nodes show exposures, CMV prediction, and breadth of rhinoviral antibodies (N_rhinovirus). Edges bind exposures with 1. mediators (blue), representing deconvoluted cell proportions, and 2. outcomes (red), representing TLs. Mediation effects are visualized as edges binding exposures with mediators and mediators with outcomes.

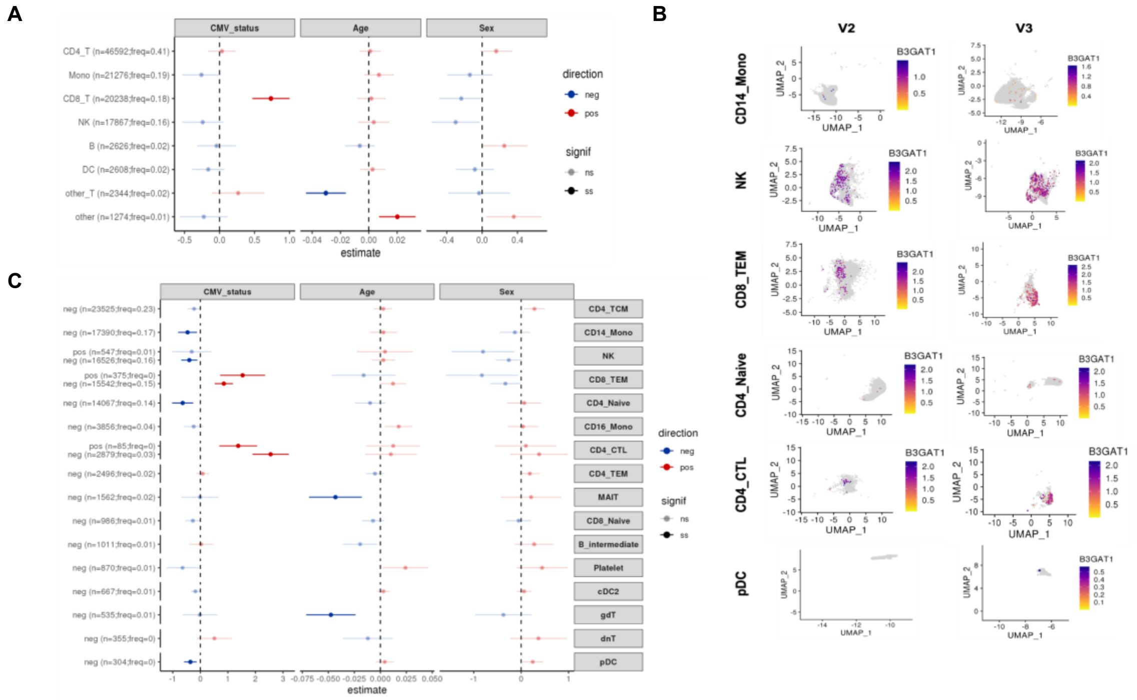

Figure S2. Cellular composition changes associated to CMV seropositivity, related to Figure 4. A. Forest plot showing the linear association between CMV serostatus, age or sex, and low-level (Azimuth's l1) cell-type-proportions from the Oelen2022 scRNA-seq data. Estimated effect values accounting for both biological (sex and age) and technical (10X Chromium Single Cell 3' chemistry and experimental batch) covariates are displayed. Error bars represent the 95% confidence interval of the estimated effect. Color indicates the sign of the estimated effect values (red: positive, blue: negative). Shading represents the statistical significance of the estimated effect values (darker: significant, FDR ≤ 0.05; lighter: non-significant, FDR > 0.5). The absolute number and relative frequency of each cell type is shown. **B.** UMAPs showing the expression of *B3GAT1* (CD57-encoding gene) in each of the high-level (Azimuth's l2) cell types altered by CMV seropositivity in (C). **C.** Forest plot showing the linear association between CMV serostatus, age or sex, and high-level (Azimuth's l2) cell-type-proportions from the Oelen2022 scRNA-seq data, distinguishing *B3GAT1*+/- populations. Estimated effect values accounting for both biological (sex and age) and technical (10X Chromium Single Cell 3' chemistry and experimental batch) covariates are displayed. Error bars represent the 95% confidence interval of the estimated effect. Color indicates the sign of the estimated effect values (red: positive, blue: negative). Shading indicates the statistical significance of the estimated effect values (darker: significant, FDR ≤ 0.05; lighter: non-significant, FDR > 0.5). The absolute number and relative frequency of each cell type is shown.

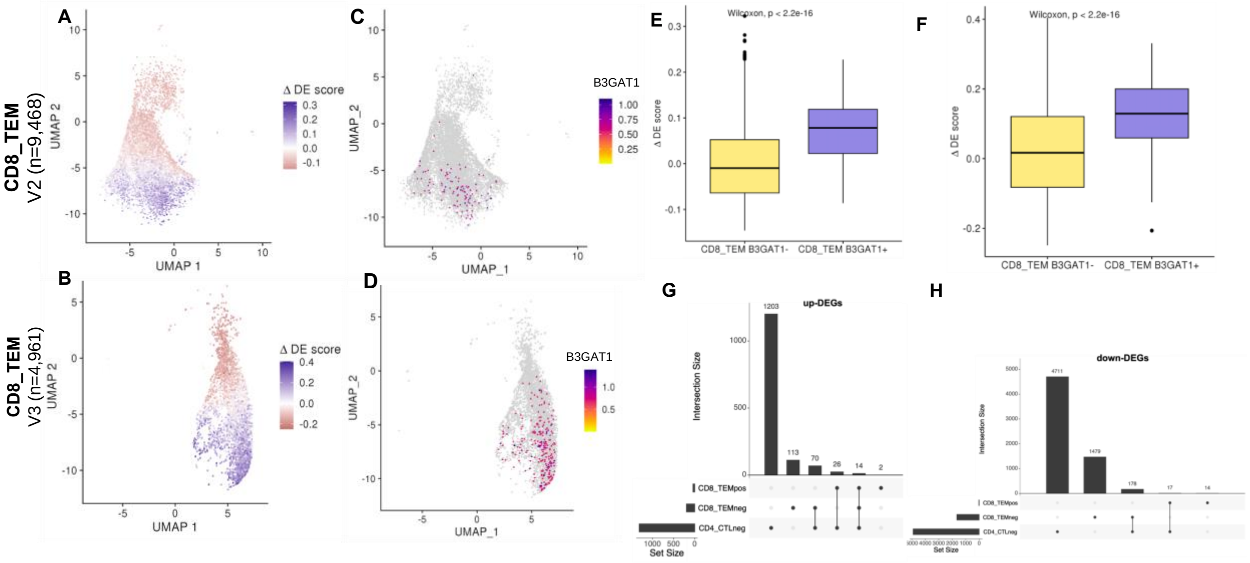

Figure S3, related to Figure 4. A–B UMAPs showing the ΔDE (differential expression) scores using the Oelen2022 scRNA-seq dataset: CD8+ TEM cells in V2 (**A**) and V3 (**B**) data. **C–D**. UMAPs showing the expression of *B3GAT1* (CD57-encoding gene) in CD8+ TEM cells using the Oelen2022 scRNA-seq V2 (**C**) and V3 (**D**) data. **E–F.** Boxplots showing the differences between the distribution of ΔDE scores in CD8+ TEM B3GAT1+ and CD8+ TEM B3GAT1- cells using the Oelen2022 scRNA-seq V2 (**E**) and V3 (**F**) data. **G–H.** Upset plots showing the number of up-regulated (**G**) and down-regulated (**H**) DEGs shared or unique among the high-level (Azimuth's l2) B3GAT1+/- cell types using the Oelen 2022 scRNA-seq data. 'pos' and 'neg' after the l2 cell-type refer to 'B3GAT1+' and 'B3GAT1'-', respectively.
